# Postnatal refinement of Ca_V_ channel isoform composition, electrical activity, and insulin release in mouse pancreatic β-cells

**DOI:** 10.1080/19336950.2026.2732550

**Published:** 2026-09-15

**Authors:** Tamara Theiner, Noelia Jacobo-Piqueras, Dorina Tota, Petronel Tuluc

**Affiliations:** Department of Pharmacology and Toxicology, Institute of Pharmacy, University of Innsbruck, Innsbruck, Austria

**Keywords:** Pancreatic β-cells, voltage-gated calcium channel, Ca_V_, development, insulin release

## Abstract

Pancreatic β-cell mass and glucose-stimulated insulin secretion strongly increase during postnatal development. Voltage-gated calcium channels (Ca_V_) are the key determinants of pancreatic β-cell electrical activity, insulin granule exocytosis, β-cell proliferation and survival. Here we show that β-cells of 1-day-old mice have significantly smaller Ca_V_ currents compared to 14-day (juvenile) or 3-month-old (adult) mice, as well as a different Ca_V_ isoform composition. In neonates, P/Q-type channels contribute ~53% of the whole-cell current while L-type channels contribute only ~25%. The reduced L-type current is associated with a complete absence of β-cell-specific glucose-induced excitability even though the β-cells depolarize at a lower glucose threshold. By postnatal day 14, the L-type contribution increases to ~44%, reaching ~50% in adults, and P/Q-type currents decrease to ~20%. Nevertheless, the total β-cell calcium influx density is largest in juvenile mice consistent with a lower-threshold glucose dependence of the electrical activity compared to adults. Despite the larger calcium influx, the islets of 14-day-old mice show significantly reduced vesicle exocytosis, insulin release and insulin content. Our data show extensive postnatal remodeling in β-cell mass and glucose sensitivity, and an age-dependent refinement of Ca_V_ isoform composition, electrical activity, and insulin release. The cumulative increase in β-cell mass and insulin release causes a ~ 7-fold increase in plasma insulin levels between 14-day- and 3-month-old mice.

## ARTICLE HIGHLIGHTS

- Postnatal β-cell development involves a significant increase in β-cell mass and glucose-stimulated insulin secretion.
- The contribution of P/Q-type currents to total calcium influx decreases from 53% to 20% during postnatal development, while L-type contribution increases progressively from 25% to 50%.
- Neonatal β-cells show a complete absence of the characteristic glucose-induced electrical activity while juvenile β-cells show reduced vesicle exocytosis and insulin release despite larger calcium influx.
- Developmental regulation of Ca_V_ isoform composition alters glucose sensitivity and insulin release, offering insight into postnatal metabolic adaptations.

## Introduction

Pancreatic β-cells of the islets of Langerhans secrete insulin, the only hormone capable of lowering the plasma glucose levels. Matching the metabolic demands, β-cell glucose-dependent insulin secretion (GSIS) undergoes a rapid postnatal maturation process in both rodents [1–5] and humans [6,7]. Phenotypically, neonatal β-cells secrete insulin in response to lower extracellular glucose levels compared to mature β-cells [8], but the total amount of insulin release is substantially smaller in young islets compared to adults [8,9]. While the reduced insulin secretion could be attributed to reduced hormone synthesis or an altered vesicle exocytosis complex [10], the different sensitivity of glucose-induced electrical activity during postnatal development depends on the ion channel composition. The β-cell glucose-dependent membrane depolarization is controlled by ATP inhibition of the K_ATP_ potassium channels [11–13]. Consequently, previously it has been shown that K_ATP_ channel functional expression [14] and ATP regulation are reduced in fetal or neonatal β-cells [2]. However, while K_ATP_ channel glucose-dependent inhibition allows a membrane depolarization, the initiation of the electrical activity and insulin granule exocytosis is dependent on the activation of the voltage-gated calcium channels (Ca_V_) [13].

The β-cells of mice and humans express several Ca_V_ isoforms with distinct roles in β-cell function [13,15,16]. Under normal physiological conditions, adult mouse β-cells do not express T-type calcium channels [17,18]. Previously it has been shown that in adult β-cells of both mouse [19] and humans ~20% of the calcium influx is conducted by Ca_V_2.1 P/Q-type channels [20,21] and 20–25% by Ca_V_2.3 R-type channels [22,23] that contribute to maintained 2^nd^ phase vesicle exocytosis [23–25]. Nevertheless, the major contributor to excitability and vesicle exocytosis in adult mouse β-cells is the L-type calcium current as demonstrated by the fact that Ca_V_1.2 deletion caused a substantial impairment of first-phase insulin release and reduced glucose-induced electrical activity [19]. Based on the previous observations that glucose-induced excitability and vesicle release are different during postnatal development [8,9,14], we hypothesized that these changes are caused by altered β-cell Ca_V_ channel isoform expression. To test this hypothesis, here we quantified the β-cell Ca_V_ channel isoform composition, β-cell glucose-induced electrical activity and insulin release in pancreatic islets of 1-day-old, 14-day-old and 3-month-old mice.

## Methods

### Mice

All experiments were performed on C57BL/6N mice. For the experiments performed on 1-day and 14-day-old mice, we did not check the sex of the animals. For the experiments performed on 3-month old animals, only male mice were used. The mice were maintained at the Specific pathogen free animal facility of the Medical University of Innsbruck under standard housing conditions with food and water available *ad libitum* on a 12 h light/dark cycle. All experiments were performed in conformity with international laws and were approved by the Austrian Ministry of Science, Research and Economy (BMWFW-66.008/0015).

### Isolation of pancreatic islets and β-cells

Pancreatic islets were enzymatically dissociated using collagenase injected via the pancreatic duct [22,26]. For electrophysiology experiments the islets were cultured overnight at 37°C and 5% CO_2_ in RPMI1640 medium (11 mM glucose) supplemented with 10% FCS, 2 mM L-glutamine, 100 μg/mL streptomycin, and 100 IU/mL penicillin.

### Immunohistochemistry

The dissected pancreas was fixed with 4% paraformaldehyde (~8 h), dehydrated overnight at 4°C in 30% sucrose, frozen in Tissue-Tek O.C.T (VWR, 4583), sliced at 15 μm thickness using a cryostat (Leica, CM1860) at −18°C. The pancreatic slices were labeled using the guinea-pig anti-insulin (Dako, A0564, 1:1000) and mouse anti-glucagon (Sigma, G2654, 1:1000) detected by goat anti guinea-pig IgG H&L (Abcam, ab175678, Alexa Fluor® 405, 1:4000) and goat anti-mouse IgG H&L (Invitrogen, A11031, Alexa Fluor® 568, 1:4000). The cell proliferation was identified by immunolabelling the Ki67 protein (rb-anti-Ki67, Abcam, ab16667, 1:1000), goat anti-rabbit IgG H&L (Invitrogen, A11008, Alexa Fluor® 488, 1:4000).

### Electrophysiology

The calcium and potassium currents, and electrical activity were measured in β-cells within intact islet using the perforated patch-clamp technique by adding 240 μg/mL amphotericin B (Merck, Y0000005) in the pipette solution. The voltage or current signals were acquired and digitized using a HEKA EPC10 amplifier controlled by Patchmaster software. The increase in cell capacitance was recorded in β-cells in intact islets bathed at 32°C–33°C using the rupture-patch configuration. The capacitance increase was measured using the lock-in and sine+DC mode of the HEKA amplifier. The readily releasable pool (RRP) was estimated as the increase in membrane capacitance (ΔCm) evoked by the first depolarizing step of the train, and the total releasable pool as the cumulative ΔCm after the full train of 10 steps. For calcium current recordings the pipette solution contained (in mM) 76 Cs_2_SO_4_, 10 CsCl, 10 KCl, 1 MgCl_2_, 5 HEPES (pH 7.2 with CsOH). For the capacitance measurements, the pipette solution contained (in mM) 140 CsCl, 10 NaCl, 1 MgCl_2_, 0.05 EGTA, 5 HEPES, 0.1 cAMP, and 0.1 MgATP (pH 7.2 with CsOH). The extracellular solution for recording calcium currents and capacitance change contained (in mM) 20 TEA-Cl, 120 NaCl, 3.6 KCl, 2 NaHCO_3_, 0.5 NaH_2_PO_4_, 0.5 MgSO_4_, 5 HEPES, 2.6 CaCl_2_, 5 glucose (pH 7.4 with NaOH). The bath solution when measuring the glucose-induced electrical activity or K_ATP_ currents contained (in mM) 140 NaCl, 3.6 KCl, 0.5 NaH_2_PO_4_, 0.5 MgSO_4_, 2.5 CaCl_2_, 2 NaHCO_3_, 5 HEPES (pH 7.4) supplemented with different glucose concentrations as indicated in the figures. The pipette solution contained (in mM) 76 K_2_SO_4_, 10 NaCl, 10 KCl, 1 MgCl_2_, 5 HEPES (pH 7.35 with KOH). The voltage dependence of sodium current inactivation was used to distinguish between α- and β-cells [22,27].

### Dynamic insulin release and insulin content

After dissection and 24 h recovery in RPMI1640 medium (11 mM glucose), the islets were perfused with a physiological solution containing (in mM) 140 NaCl, 3.6 KCl, 2 NaHCO_3_, 0.5 NaH_2_PO_4_, 0.5 MgSO_4_, 5 HEPES, and 2.5 CaCl_2_ with different glucose concentrations (2, 5, 7.5, 10, 15 mM) at 37°C. The flow rate was set to 200 μL/min and the fractions collected every 2 minutes. Insulin concentration in the 2 minute fractions was measured with a sandwich ELISA (Abcam, ab8304 and ab28063). To exclude differences in islet size, the insulin release per islet and unit of time was calculated based on islet area and flow rate respectively as we have done previously [28].

### Statistical analysis

Data analysis was performed using FitMaster and Patchmaster (HEKA electronics, version 2 × 92), Clampfit 10.7, Sigma Plot 13.0, GraphPad Prism8, Origin 2020, Excel 2016 (Microsoft), and ImageJ. All data are presented as mean ± SEM for the indicated number (n) of experiments. Statistical significance was evaluated using one-way ANOVA, two-way ANOVA, unpaired Student’s t-test or Wilcoxon-Mann-Whitney-Test.

## Results

### In neonatal and 14-day-old mice the islets are smaller but show a higher proliferation rate

**Figure 1.**
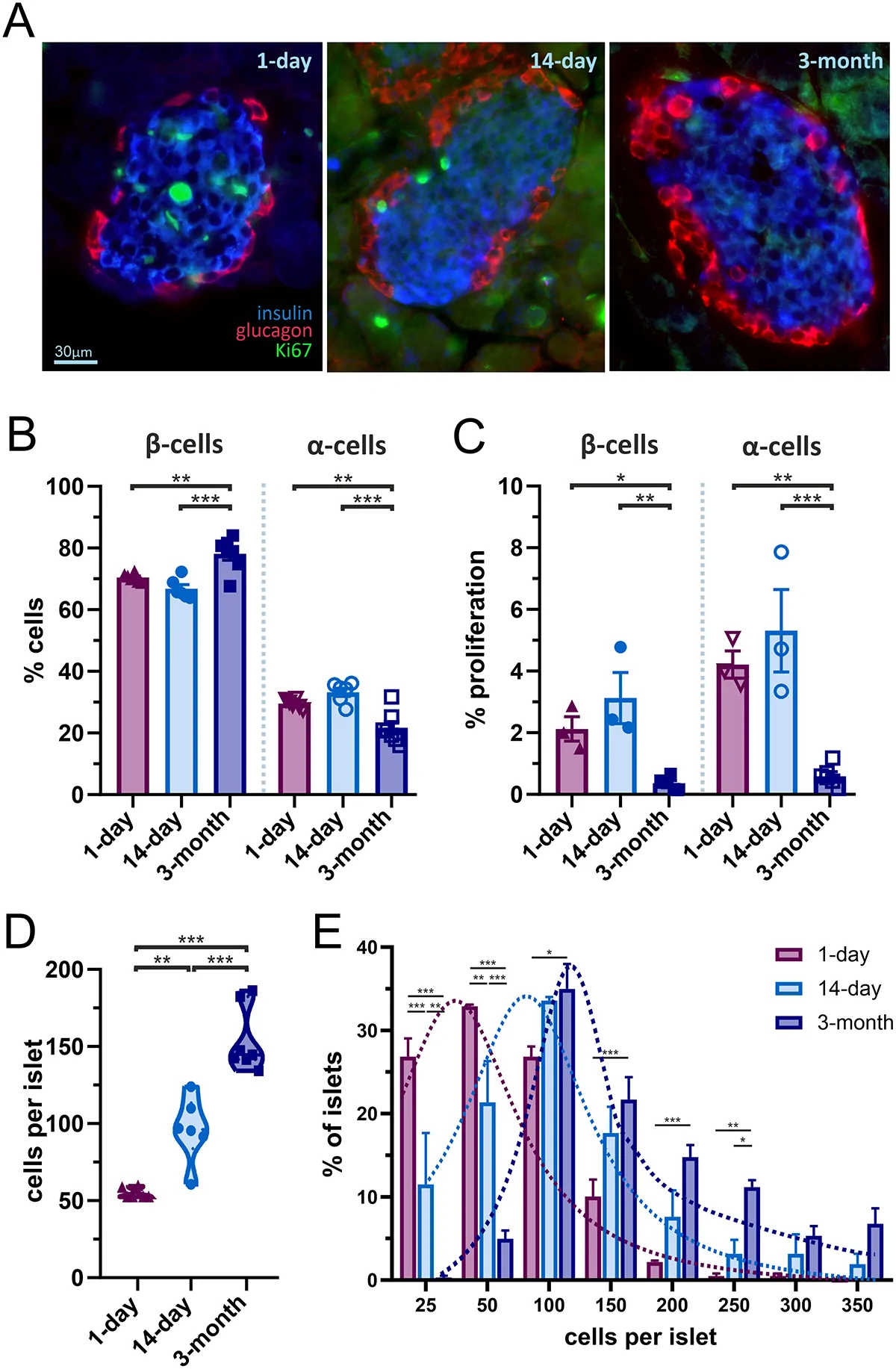
Pancreatic islets of neonatal and 14-day-old mice are smaller in size but show higher cell proliferation compared to 3-month-old. (A) Immuno-staining of pancreatic slices from 1-day, 14-day and 3-month-old mice. Pancreatic β-cells (blue) occupy the islet core, while α-cells (red) form a mantle in the periphery. The proliferating cells positive for Ki67 are labeled in green. (B) Percentage of α- and β-cells (1-day α-cell = 29.57 ± 0.57%, β-cell = 70.42 ± 0.57%, *N* = 6 mice), 14-day (α-cell = 33.22 ± 1.31%, β-cell = 66.77 ± 1.31%, *N* = 6 mice), 3-month (α-cell = 21.69 ± 8.39%, β-cell = 78.09 ± 8.42%, *N* = 7 mice). (C) Percentage of Ki67-positive proliferating α- and β-cells (1-day α-cells = 4.21 ± 0.44%, β-cell = 2.12 ± 0.39%, *N* = 3; 14-day α-cell = 5.31 ± 1.33%, β-cell = 3.12 ± 0.82%, *N* = 3; 3-month α-cell = 0.58 ± 0.14%, β-cell = 0.36 ± 0.07%, *N* = 6). (D) Average islet size for each tested age (1-day *N* = 54.63 ± 1.41 cells/islet; 14-day *N* = 96.39 ± 8.66 cells/islet; 3-month *N* = 154.13 ± 7.94 cells/islet). (E) Distribution of islet size (1-day *N* = 6, 14-day *N* = 6, 3-month *N* = 7 mice; >60 islets each). Error bars represent SEM. Statistical analysis with one-way ANOVA or two-way ANOVA followed by Bonferroni post-hoc test: *p < 0.05; **p < 0.01; ***p < 0.001.

In pancreatic sections of 1-day, 14-day, and 3-month-old mice the islets showed a well-structured morphology with β-cells (blue) forming the center of the islet and α-cells (red) forming a mantel (Figure 1(A)). Nevertheless, the islet size and composition substantially change during postnatal development. The islets of 3-month-old mice showed an ~8% larger contribution of β-cells to total islet cell count compared to 1-day-old (*p* = 0.002) and ~11% compared to 14-day-old mice (*p* = 0.001) (Figure 1(B)). The α-cell contribution increased by ~3% from 1-day to 14-day-old (*p* = 0.007) but decreased by ~8% to adults (*p* = 0.004). The α-cell proliferation rate was ~7-fold higher in 1-day-old and ~9-fold higher 14-day-old compared to adults (Figure 1(C)). Similarly, the β-cell proliferation was ~6-fold higher in 1-day-old and ~9-fold higher in juveniles compared to adults. Consequently, the islet size increased by ~76% from 1-day to 14-day (*p* = 0.022) and showed a further ~60% increase from 14-day to 3-month (*p* < 0.0001) (Figure 1(D,E)). Since calcium influx is a crucial determinant of β-cell proliferation and survival [28–40], we hypothesized that the changes in islet architecture and β-cell proliferation correlate with different Ca_V_ currents.

### Voltage-gated calcium currents have different biophysical properties during postnatal development

**Figure 2.**
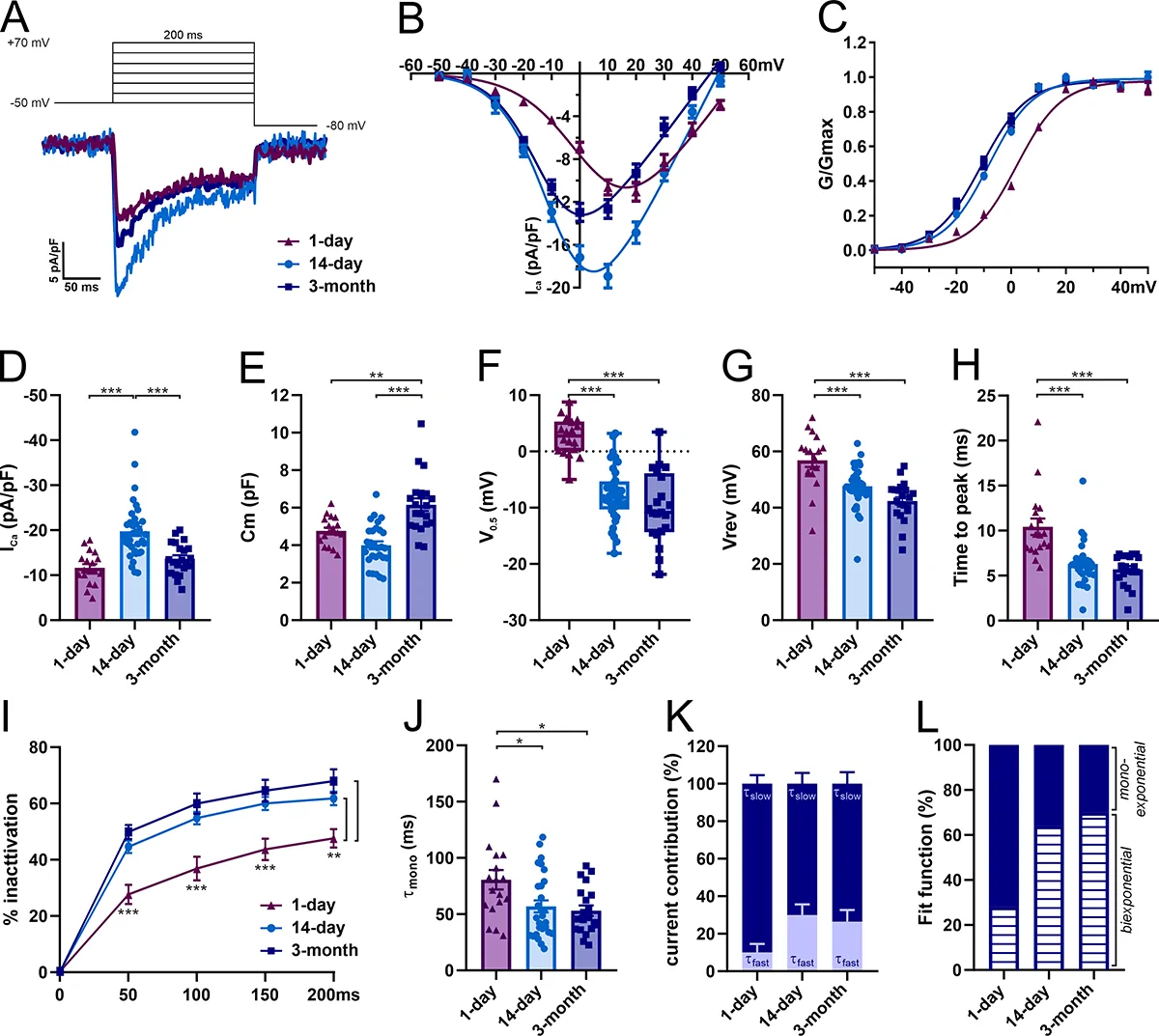
Voltage-gated calcium currents have different biophysical properties during postnatal development. (A) Representative maximum whole-cell calcium currents recorded from β-cells in intact pancreatic islets. (B) The voltage dependence of the calcium current (I-V) in the β-cells of the three tested ages (1-day *n* = 18; 14-day *n* = 37; 3-month *n* = 21; at least 3 mice each condition). (C) Normalized voltage-dependence of activation. (D) Maximum current amplitude normalized to cell capacitance (1-day I_peak_ =  −11.65 ± 0.82 pA/pF; 14-day I_peak_ =  −19.80 ± 1.01 pA/pf; 3-month I_peak_ =  −13.64 ± 0.78 pA/pf). (E) Cell capacitance measured at −80 mV holding potential (1-day C_m_ = 4.76 ± 0.17 pF; 14-day C_m_ = 4.17 ± 0.21 pF; 3-month C_m_ = 6.16 ± 0.33 pF). (F) Voltage-dependence of calcium influx half maximal activation (V_0.5_) (1-day V_0.5_ = 2.59 ± 0.77 mV; 14-day V_0.5_ =  −7.47 ± 0.82 mV; 3-month V_0.5_ =  −9.95 ± 1.36 mV). (G) Extrapolated reversal potential estimated from the I-V fit (1-day V_rev_ = 56.82 ± 2.24 mV; 14-day V_rev_ = 47.62 ± 1.20 mV; 3-month V_rev_ = 42.39 ± 1.52 mV). (H) Quantification of calcium influx activation kinetics measured by the time to peak (TTP) (1-day TTP = 10.43 ± 0.91 ms, *n* = 18; 14-day TTP = 6.30 ± 0.43 ms, *n* = 31; 3-month TTP = 5.69 ± 0.38 ms, *n* = 20). (I) Fractional current inactivation @50 ms, @100 ms, @150 ms, and @200 ms measured at maximum calcium influx (1-day *n* = 18; 14-day *n* = 31; 3-month *n* = 20; at least 3 mice each condition). (J) Time constant of the single exponential fit of current inactivation (1-day τ_mono_ = 80.54 ± 8.44 ms; 14-day τ_mono_ = 56.91 ± 5.41 ms; 3-month τ_mono_ = 53.09 ± 4.73 ms). (K) Contribution of the fast and slow inactivation components characterized by their specific τ_slow_ and τ_fast_ time constants present in a fraction of cells. (L) Percentage of cells that can be described only by a mono-exponential (solid blue) (1-day *n* = 18; 14-day *n* = 28; 3-month *n* = 20; at least 3 mice each condition) or a bi-exponential (blue-white striped) (1-day *n* = 5; 14-day *n* = 18; 3-month *n* = 14; at least 3 mice each condition) function. Error bars represent SEM. Statistical analysis with one-way ANOVA or two-way ANOVA followed by Bonferroni post-hoc test: *p < 0.05; **p < 0.01; ***p < 0.001.

To test if β-cell high voltage-gated Ca_V_ currents have different biophysical properties during postnatal development, we performed voltage-clamp experiments in β-cells within intact isolated islets and measured the calcium influx in response to step membrane depolarization from −50 mV to +70 mV in 10 mV increments. Prior to the protocol, a 1 second conditioning pulse to −50 mV was applied to inactivate the voltage-gated sodium channels (Na_V_) and T-type Ca_V_ channels. Sample maximum current influx (Figure 2(A)) and average voltage-dependence of calcium influx (Figure 2(B,D)) demonstrate that calcium current density is ~41% smaller in β-cells of 1-day-old compared to 14-day (*p* < 0.0001) but ~31% higher in 14-day-old compared to adults (*p* < 0.0001). The absolute calcium current amplitude was significantly smaller in β-cells of neonatal mice (I_peak_ = −55.61 ± 4.43 pA, *p* < 0.0005) compared to the two later developmental stages while the cell capacitance was similar compared to 14-day-old mice. However, the absolute peak current amplitude was similar in juvenile (I_peak_ = −78.80 ± 3.58 pA) and adult mice (I_peak_ = −82.31 ± 5.40 pA) but because the cell capacitance (Figure 2(E)) was ~47% larger in adults (*p* < 0.001) compared to juveniles, the current density was significantly larger in juveniles. The voltage dependence of activation (Figure 2(C,F)) did not show a significant difference between adult and juvenile β-cells, but was shifted by ~7 mV toward more depolarized potential in neonates compared to adults (*p* < 0.0001). Consequently, in neonatal β-cells the extrapolated reversal potential (V_rev_) was shifted toward more depolarized potentials compared to 14-day and 3-month (Figure 2(G)). Additionally, the time necessary for the calcium influx to reach the maximum amplitude (Figure 2(H)) was longer in neonatal β-cells compared to 14-day-old or adults (*p* < 0.0001). Similarly, the fractional current inactivation was significantly lower in neonatal β-cells, compared to later developmental stages (Figure 2(I)). Pancreatic β-cells typically show a biphasic calcium current inactivation, with an initial fast and a subsequent slow component [41]. While in 70–80% of β-cells isolated from adult and 14-day-old mice the calcium current inactivation could be fitted by a double exponential function, only ~20% β-cells from 1-day-old had similar properties (Figure 2(L)). Furthermore, the fast component (A_fast_) accounted for ~30% of total current in 14-day and 3-month-old mice but only 10% in β-cells of neonatal mice (Figure 2(K)). Due to this discrepancy, we fitted the calcium current inactivation of all cells and developmental stages with a mono-exponential function. The time constants of inactivation (Figure 2(J)) were similar between 14-day and 3-month-old, but ~42% slower for calcium currents of neonatal β-cells (*p* = 0.02 compared to 14-day). Cumulatively, this demonstrates different calcium current biophysical properties in β-cell during postnatal development, suggesting a different Ca_V_ isoform composition.

### β-cell Ca_V_ channel isoform composition changes during postnatal development

**Figure 3.**
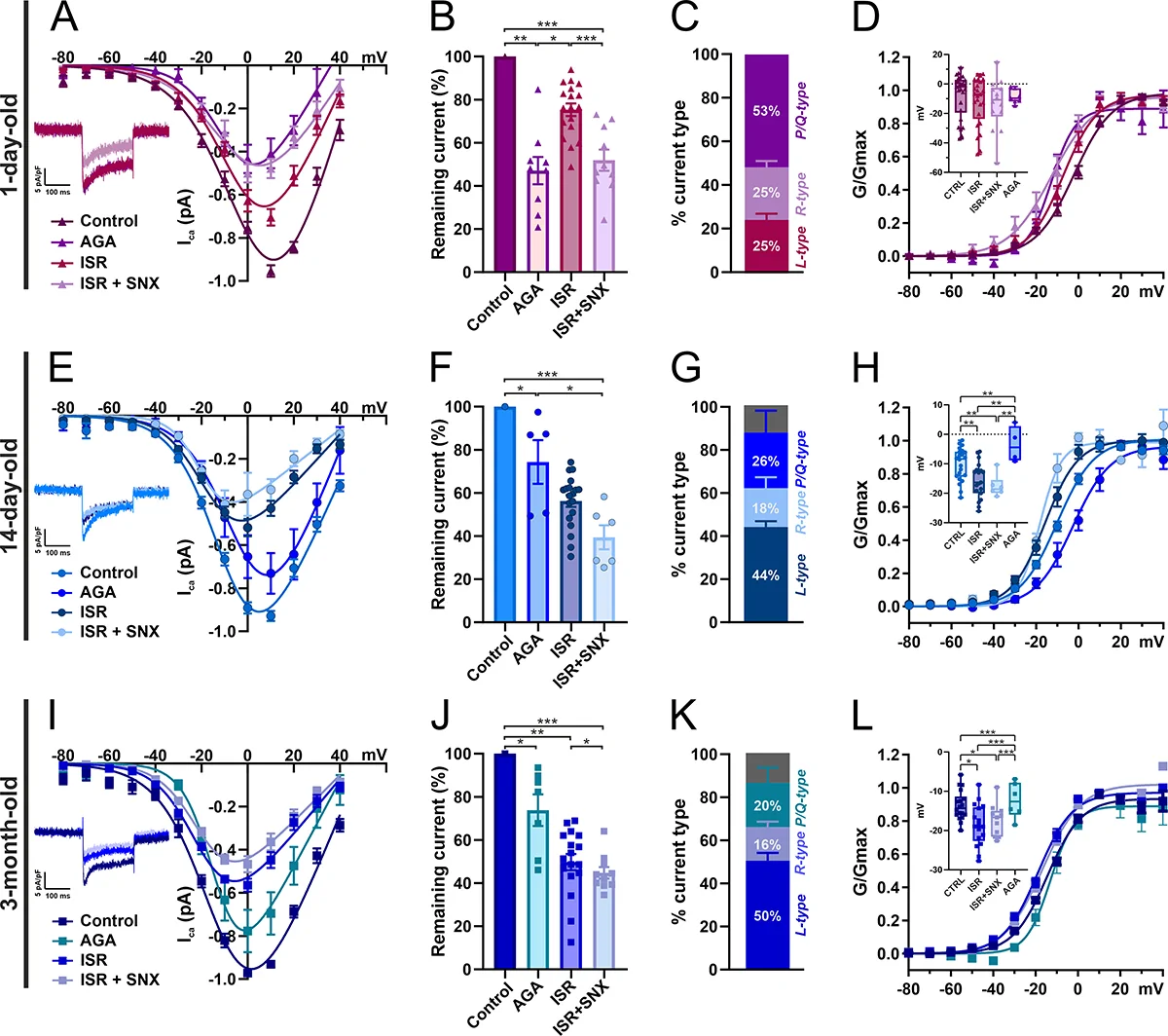
β-cell Ca_V_ isoform composition changes during postnatal development. (A, E, I) Voltage dependence of calcium currents in β-cells of islets from 1-day, 14-day and 3-month-old mice before and after application of 2 μM isradipine (ISR) or 2 μM ISR +100 nM SNX-482 or 200 nM ω-Agatoxin-IVA (aga). Sample traces in the insets. (B, F, J) Percentage of calcium current remaining after 2 μM isradipine (ISR), 2 μM ISR +100 nM SNX-482, or 200 nM ω-Agatoxin-IVA (aga) (1-day control *n* = 28; ISR = 75.31 ± 2.84%, *n* = 19; SNX = 51.88 ± 4.65%, *n* = 10, aga = 47.08 ± 6.35%, *n* = 9), (14-day control *n* = 23; ISR = 56.20 ± 2.69%, *n* = 19; SNX = 39.39 ± 5.08%, *n* = 6; aga = 74.34 ± 10.11%, *n* = 5), (3-month control *n* = 19; ISR = 49.72 ± 3.62%, *n* = 17; SNX = 44.71 ± 2.52%, *n* = 10, aga = 79.69 ± 7.36%, *n* = 8). (C, G, K) Calculated contribution of the P/Q-type, L-type, and R-type current component in the β-cells of the three tested ages (1-day L-type = 24.68 ± 2.84%, R-type = 24.29 ± 2.90%, P/Q-type = 52.91 ± 6.35%; 14-day L-type = 43.79 ± 2.68%, R-type = 17.97 ± 5.02%, P/Q-type = 25.66 ± 10.11%; 3-month L-type = 50.27 ± 3.62%, R-type = 15.6 ± 2.41%, P/Q-type = 20.30 ± 7.13%). (D, H, L) Voltage-dependence of conductance in the presence and absence of the different Ca_V_ blockers (1-day, control V_0.5_ = 0.26 ± 1.39 mV, ISR V_0.5_ =  −4.07 ± 2.05 mV, SNX V_0.5_ =  −7.47 ± 3.80 mV, aga V_0.5_ = 1.29 ± 1.82 mV; 14-day control V_0.5_ =  −10.12 ± 1.15 mV, ISR V_0.5_ =  −15.66 ± 1.35 mV, SNX V_0.5_ =  −17.54 ± 1.41 mV, aga V_0.5_ = 6.51 ± 3.06 mV; 3-month control V_0.5_ =  −13.23 ± 0.85 mV, ISR V_0.5_ =  −17.29 ± 1.29 mV, SNX V_0.5_ =  −17.00 ± 1.34 mV, aga V_0.5_ =  −2.31 ± 1.84 mV). Error bars represent SEM. Statistical analysis with one-way ANOVA followed by Bonferroni post-hoc test: *p < 0.05; **p < 0.01; ***p < 0.001, comparison between the indicated conditions for each group.

To identify which Ca_V_ isoforms are expressed in β-cells during postnatal development we measured calcium influx in the presence or absence of the specific L-type calcium channel blocker isradipine (ISR, 2 μM), ISR plus the specific R-type Ca_V_2.3 channel blocker SNX-482 (SNX, 100 nM), or the specific P/Q-type blocker ω-Agatoxin-IVA (AGA, 200 nM) [42–44]. Sample traces of control, ISR and ISR+SNX conditions are presented in Figure 3(A,E,I) insets. To exclude potential isoform-specific voltage-dependent steady-state inactivation [26], we measured the calcium influx starting from a resting membrane potential of −80 mV. In β-cells of 1-day-old mice application of 2 μM ISR significantly reduced β-cell calcium influx by ~25% (*p* = 0.0089) while the subsequent application of SNX-482 further reduced the total calcium current influx by ~25% (*p* = 0.0069). Surprisingly, the application of 200 nM ω-Agatoxin-IVA caused a significant reduction in current amplitude by ~53% (*p* = 0.0045) (Figure 3(A–C)). The pharmacological inhibition of any calcium currents did not cause a significant change in the voltage dependence of activation (Figure 3(D) conductance curve, and inset V_0.5_). In ~54% of the cells (13 from 24 cells) we also observed a fast and transient T-type calcium current with the maximum amplitude at −30 mV that could not be blocked neither by ISR nor by SNX-482. In juvenile β-cells ISR caused a ~ 44% inhibition of calcium influx (*p* < 0.0001) while SNX caused an additional ~18% reduction (*p* = 0.0234) (Figure 3(E–G)). ISR application also significantly shifted by ~5 mV the voltage dependence of activation toward more hyperpolarized potentials (*p* < 0.01) while SNX application did not cause an additional shift (Figure 3(H) conductance curve, and inset V_0.5_). ω-Agatoxin-IVA application caused a ~ 26% reduction in current amplitude (*p* = 0.0347) and induced a significant shift in the voltage dependence of activation by ~17 mV toward more depolarizing potentials (*p* = 0.0084). In adult β-cells, ISR application reduced the calcium current amplitude by ~50% (*p* < 0.0001) and SNX-482 reduced the calcium currents by an additional ~16% (*p* = 0.002) (Figure 3(I–K)). The voltage-dependence of activation was significantly shifted toward lower membrane potentials by ISR application by ~4 mV (*p* = 0.01) but was not further affected by SNX application (Figure 3(L) conductance curve, and inset V_0.5_). The addition of 200 nM ω-Agatoxin-IVA to the bath solution induced a 20% reduction in the calcium current amplitude (*p* = 0.0028) and a significant shift in the voltage dependence of activation by ~11 mV (*p* < 0.001). In summary, these results clearly indicate a different Ca_V_ isoform composition in β-cells during postnatal development.

### β-cell electrical activity and glucose sensitivity change during postnatal development

**Figure 4.**
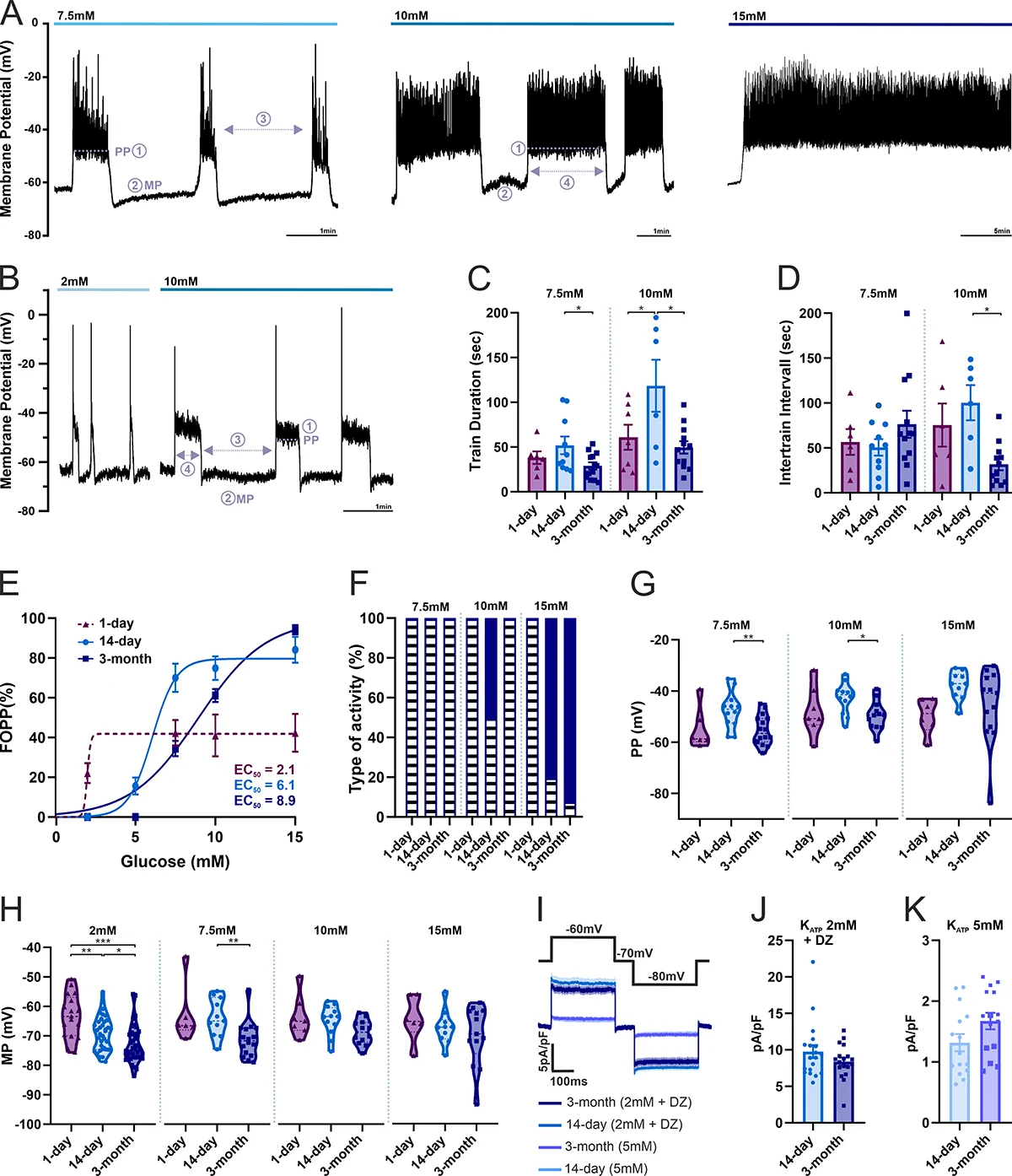
The glucose sensitivity of the electrical activity changes during postnatal development. (A) Representative current-clamp recordings of glucose-induced electrical activity of β-cell in intact islet isolated from 3-month old mice stimulated with 7.5 mM, 10 mM and 15 mM glucose. In the representative trace, ① pp – plateau potential, ② MP – membrane potential, ③ the duration between trains, ④ the train duration. (B) Exemplary recording of electrical activity induced by 2 mM and 10 mM glucose recorded in a β-cell of an islet isolated from a 1-day old mouse. (C) Average duration of the AP-trains in β-cells of the three tested ages stimulated with 7.5 mM (14-day 52.03 ± 9.48 s, *n* = 10; 3-month 28.87 ± 3.88 s, *n* = 13), or 10 mM glucose (1-day 61.26 ± 14.11 s, *n* = 7; 14-day 119.51 ± 26.92 s, *n* = 6; 3-month 49.75 ± 6.74 s, *n* = 12). (D) Duration of the electrically silent periods between AP-trains (in 10 mM glucose, 14-days 100.28 ± 17.86 s; 3-month 31.75 ± 6.41 s). (E) Fraction of time spent on the depolarized plateau phase (FOPP) from the total recording time (@ 2 mM glucose: 1-day *n* = 19; 14-day *n* = 0; 3-month *n* = 0), (@ 5 mM glucose: 1-day *n* = n.A.; 14-day *n* = 10; 3-month *n* = 0), (@ 7.5 mM glucose: 1-day *n* = 6; 14-day *n* = 12; 3-month *n* = 1), (@ 10 mM glucose: 1-day *n* = 7; 14-day *n* = 11; 3-month *n* = 11), (@ 15 mM glucose: 1-day *n* = 6; 14-day *n* = 10; 3-month *n* = 14). (F) Percentage of β-cells showing a firing pattern consisting of separated AP-trains (dashed) or one continuous train (filled). Cells with continuous electrical activity longer than 3 minutes were counted as continuous firing. (G) Membrane potential during the depolarizing plateau recorded in β-cells stimulated with 7.5 mM (1-day *n* = 6; 14-day *n* = 11; 3-month *n* = 15), 10 mM (1-day *n* = 7; 14-day *n* = 12; 3-month *n* = 12), and 15 mM glucose (1-day *n* = 6; 14-day *n* = 10; 3-month *n* = 13) (@7.5 mM glucose 14-day V_m_ =  −47.79 ± 1.97 mV, 3-month V_m_ =  −55.16 ± 1.47 mV; @ 10 mM glucose 14-day V_m_ =  −42.22 ± 1.66 mV, 3-month V_m_ =  −49.12 ± 1.58 mV). (H) Average membrane potential recorded during the electrically silent periods between the AP-trains (@ 2 mM glucose 1-day V_m_ =  −63.38 ± 1.78 mV; 14-day V_m_ =  −69.23 ± 0.95 mV; 3-month V_m_ =  −73.89 ± 1.01 mV; @ 7.5 mM glucose 14-day V_m_ =  −64.17 ± 1.79 mV, 3-month V_m_ =  −71.61 ± 1.55 mV). (I) Average total K_ATP_ K^+^ currents recorded in the presence of 2 mM glucose and 340 μM diazoxide or 5 mM glucose from β-cells of 14-day and 3-month old mice using the protocol depicted above the traces (14-day *n* = 18; 3-month *n* = 18). (J) Maximum amplitude of the K_ATP_ K^+^ currents recorded in the presence of 2 mM glucose and 340 μM diazoxide. (K) Maximum amplitude of the K_ATP_ K^+^ currents recorded in the presence of 5 mM glucose. Error bars represent SEM. Statistical analysis with one-way or two-way ANOVA, followed by Bonferroni post-hoc test: *p < 0.05; **p < 0.01; ***p < 0.001.

To investigate how the differential Ca_V_ isoform expression affects the β-cell glucose-induced excitability we performed current-clamp recording in β-cells within intact islet (Figure 4). In β-cells of both 14-day and 3-month-old mice increasing the glucose concentration from 2 mM to 7.5 mM induced an increase in electrical activity composed of trains of action potentials (AP) superimposed on a depolarized plateau phase ① separated by hyperpolarized resting intervals ②. Increasing the glucose concentration caused a shortening of the silent intervals ③ and increased AP-train duration ④ culminating with a continuous AP-train in 15 mM glucose (Figure 4(A)). Surprisingly, in neonatal islets the β-cells failed to display the characteristic AP-trains (Figure 4(B)) and fired only one or two APs on top of the depolarizing plateau. Additionally, this behavior was independent of the extracellular glucose levels. None of the neonatal β-cells showed a continuous electrical activity even in 15 mM glucose (Figure 4(F)) but they displayed plateau potential depolarizations already in 2 mM glucose (Figure 4(B)), condition in which the juvenile or adult β-cell remained electrically silent. In 7.5 mM glucose the juvenile β-cells showed ~44% longer AP-train duration compared to adults (*p* < 0.05) while in 10 mM glucose the AP-train duration in juveniles was ~49% longer compared to neonates and ~58% compared to adults (*p* < 0.05) (Figure 4(C)). While in 7.5 mM glucose the inter-train interval was not significantly different in β-cells of the three tested developmental stages, in 10 mM glucose the inter-train interval was ~68% shorter in β-cells of 3-month-old compared to 14-days (Figure 4(D)). However, ~50% of pancreatic β-cells of 14-day-old mice showed a continuous AP-train already in 10 mM glucose whereas adults showed continuous AP-trains only in 15 mM glucose (Figure 4(F)). Because of the shorter AP-trains and longer inter-train intervals, the fraction of depolarized plateau potential (FOPP, Figure 4(E)) reached only ~42% in neonatal β-cells at all glucose levels above 7.5 mM. However, the glucose sensitivity of the electrical activity was significantly higher in neonates (EC_50_ = 2.10 ± 2.0 mM) than in juveniles (EC_50_ = 6.05 ± 0.35 mM), which in turn were more sensitive compared to adults (EC_50_ = 8.93 ± 0.42 mM). The progressively lower glucose threshold (left-shifted glucose dependence) correlates with higher resting membrane potentials (MP) (Figure 4H) in neonatal and juvenile β-cells as well as higher plateau potentials (PP) in 7.5 and 10 mM glucose (Figure 4G) in juvenile β-cells compared to adults. As previously reported, the differences in MP and PP could be caused by an altered K_ATP_ channel expression or modulation [2,14,45]. To test this hypothesis, we measured the K_ATP_ K^+^ currents using a step pulse protocol of ±10 mV starting from a holding potential of −70 mV (Figure 4I) in juvenile and adult β-cells where we observed the largest differences in MP and PP (Figure 4(G,H)). To measure the maximum K_ATP_ K^+^ currents we bathed the islets in 2 mM glucose plus 340 μM diazoxide, a K_ATP_ channel activator (Figure 4(J)). Under these experimental conditions, the whole-cell K_ATP_ current amplitudes were similar in β-cells of both developmental stages (Figure 4(I,J)). Increasing the glucose concentration to 5 mM caused a similar inhibition of K_ATP_ currents by ~87% in 14-day-old and ~74% in 3-month-old mice (Figure 4(I,K)). This is in line with early seminal studies that elegantly demonstrated that K_ATP_ channels during development have a lower conductance [14] and are less regulated by ATP [2]. While we did not perform a full dose-response curve of K_ATP_ channel regulation by glucose, we can say that at least in 5 mM glucose the increased β-cell glucose-dependent excitability in juveniles is not caused by alterations in K_ATP_ K^+^ currents amplitude or modulation. In neonatal β-cells we did not record K_ATP_ currents because the immaturity of the neonatal K_ATP_-dependent metabolic coupling is already very well documented [14,16,46–49].

### Islets of 3-month-old mice secrete significantly more insulin compared to 14-days-old

**Figure 5.**
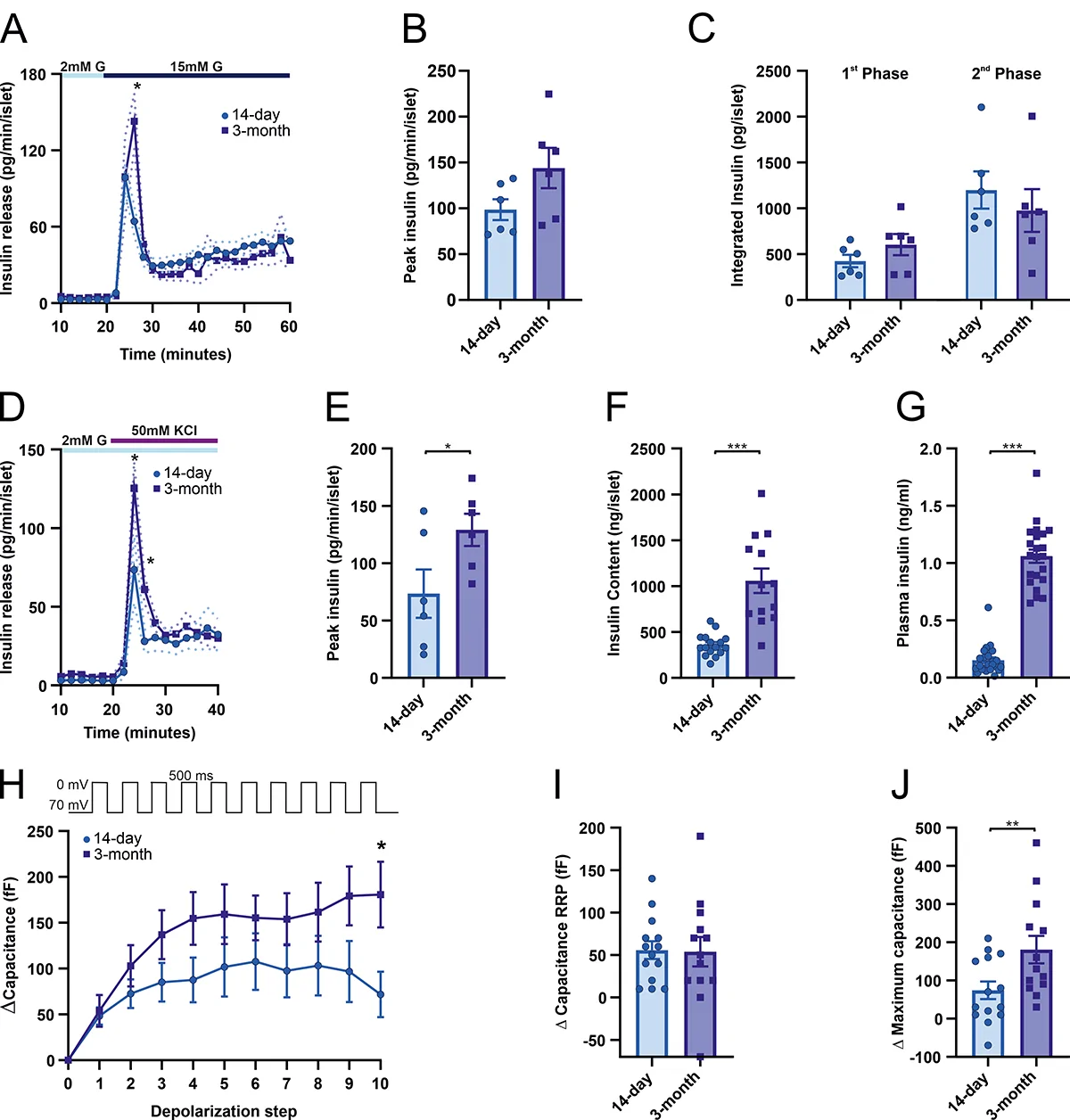
Islets of 14-day old mice release less insulin compared to islets of 3-month-old mice. (A) Average insulin secretion from 20 perfused pancreatic islets stimulated by 15 mM glucose and fractions collected every two minutes (14-day *n* = 6; 3-month *n* = 6) (@ 4 minutes 14-day 64.23 ± 11.62 pg/min/islet, 3-month 142.89 ± 21.79 pg/min/islet). (B) Maximum peak of the 1^st^ phase of insulin release. (C) Area under the curve of the 1^st^ and 2^nd^ phase insulin release stimulated by 15 mM glucose. (D) Insulin secretion from 10 perfused pancreatic islets stimulated by 50 mM kcl (14-day *n* = 6; 3-month *n* = 6). (E) Maximum peak of insulin secretion caused by 50 mM kcl stimulation (14-day 73.44 ± 21.12 pg/min/islet; 3-month 125.37 ± 16.83 pg/min/islet). (F) Insulin content of 10 freshly isolated pancreatic islets (14-day 360.25 ± 30.22 ng/islet, *n* = 16; 3-month 1058.28 ± 133.91 ng/islet, *n* = 13). (G) Plasma insulin levels in blood samples collected after decapitation from mice kept under standard housing conditions and without fasting or glucose challenge (14-day 0.15 ± 0.02 ng/mL, *n* = 24; 3-month 1.06 ± 0.05 ng/mL, *n* = 22). (H) β-cell capacitance increase during a train of 10 depolarizing steps 500 ms long from −70 mV to 0 mV (14-day *n* = 14; 3-month *n* = 13). (I) Increase in the cell capacitance caused by the first depolarizing step corresponding to the exocytosis of the readily releasable pool (RRP) of vesicles. (J) Maximum increase in cell capacitance after 10 depolarizing steps (14-day ΔCm = 74.29 ± 22.83 fF, 3-month ΔCm = 180.77 ± 35.85 fF). Error bars represent SEM. Statistical analysis with two-way ANOVA followed by Bonferroni post-hoc test and unpaired Student’s t-test: *p < 0.05; **p < 0.01; ***p < 0.001.

If pancreatic β-cells of 14-day-old mice have a larger calcium influx (Figure 2) and increased glucose-dependent electrical activity with a better glucose sensitivity (Figure 4(E,F)), do they also secrete more insulin? Step increase in glucose concentration from 2 mM to 15 mM elicited the characteristic biphasic insulin release from islets of both ages (Figure 5(A)). The maximum amplitude of the 1^st^ phase insulin release was ~2-fold smaller in 14-day-old mice 4 minutes after the initiation of the release compared to adults (*p* = 0.013). However, because the release from 14-day-old islets reached its maximum 2 minutes faster compared to adult islets, the maximum insulin release was not significantly different (Figure 5(B)). The islets of both ages also displayed a similar integral of the 1^st^ and 2^nd^ phase release (Figure 5(C)). However, the glucose-induced insulin release reports on the combined effect of glucose-dependent excitability, Ca_V_ calcium influx, vesicle exocytosis efficiency, and insulin content. To exclude the effect of different membrane excitability, we induced a direct membrane depolarization with 50 mM KCl (Figure 5(D)). Under this experimental condition the adult islets responded with ~71% higher insulin release compared to 14-day (*p* = 0.042) (Figure 5(E)). To test if vesicle exocytosis is impaired in 14-day-old β-cells despite the larger calcium currents (Figure 2(A,B,D)) we measured the increase in membrane capacitance in response to 10 steps voltage depolarization to 0 mV (Figure 5(H)). The increase in membrane capacitance following the first depolarization step representing the exocytosis of the readily releasable pool (RRP) of vesicles was similar in both tested ages (Figure 5(I)) indicating a similar coupling of calcium influx to exocytosis. However, the capacitance increases evoked by the subsequent steps remained smaller in juvenile β-cells and the maximum increase in cell capacitance after 10 steps was >2-fold smaller compared to adults (*p* = 0.02) (Figure 5(J)). While several mechanisms could be envisaged to explain the different exocytosis, the most plausible cause is a ~ 3-fold larger β-cell insulin content in 3-month-old mice (*p* < 0.001) (Figure 5(F)). Consequently, due to ~2.5-fold larger single β-cell exocytosis, ~3-fold higher insulin content, ~1.7-fold higher islet insulin release, and ~1.6-fold bigger islet size (Figure 1), the plasma insulin content (Figure 5(G)) is ~7-fold higher in adult compared to juvenile mice (*p* < 0.001).

## Discussion

Here we demonstrate that postnatal maturation of murine pancreatic β-cells is accompanied by significant changes of Ca_V_ channel isoform expression, which in turn controls the glucose sensitivity of electrical activity and insulin secretion. At the morphological level, our data show that islet architecture is established at birth, but undergoes substantial reorganization during postnatal maturation. The β-cell expansion and changes in biophysical properties we observe over the first two postnatal weeks cannot be attributed to diet, since the animals are milk-fed throughout this time window. Rather, these early changes reflect a developmentally programmed, age-dependent process in which neonatal β-cells proliferate at a high baseline rate and grow through a combination of replication of preexisting cells and ongoing neogenesis during the first postnatal weeks [50–56]. This neonatal proliferation is not driven by nutrient or glucose signaling, as suckling β-cells are refractory to the mitogenic effect of hyperglycemia and acquire glucose-responsive proliferation only later, upon weaning to a solid carbohydrate-rich diet [5,57]. Adult islets exhibit a higher β-cell fraction compared to 14-day-old or neonatal islets, accompanied by a reduction in cell proliferation. The observed decline in Ki67-positive β-cells from 1-day and 14-day-old mice to adults is consistent with previous studies showing that β-cell proliferation declines after weaning [1,3,5,9,58].

Electrophysiological analyses revealed striking developmental differences in Ca_V_ current density and biophysical properties. In β-cells from 1-day-old mice the high voltage-gated calcium currents displayed markedly smaller amplitudes and were characterized by a rightward shift in the voltage dependence of activation and slower current inactivation kinetics. The β-cells from 14-day-old mice displayed significantly larger Ca_V_ current density compared to the two other developmental stages when measured from a resting membrane potential of −50 mV. Cumulatively, this suggests significant changes in Ca_V_ channel composition during postnatal β-cell maturation. Indeed, pharmacological dissection demonstrates that neonatal β-cell have a markedly different Ca_V_ isoform composition compared to adults with the L-type and P/Q-type contributions essentially inverted. While in adult β-cells ~50% of the current is conducted by L-type channels and P/Q-type current contribute with only ~20% (Figure 5(K)), in neonatal β-cells Ca_V_2.1 channels contribute with ~53% of the current and L-type channels only with ~25% (Figure 5(C)). The R-type (Ca_V_2.3) channels show a smaller variation decreasing from ~25% in neonates, to ~18% in juveniles and ~16% in adult mice. Functionally, this reflects on the glucose-induced electrical activity and insulin release. In adult β-cells the dynamics of 1^st^ phase glucose-dependent insulin release rely on the activation of Ca_V_1.2 channels [19] and the amplitude of the 2^nd^ phase is modulated by the Ca_V_2.3 channels [23,59–61], whereas Ca_V_2.1 channels do not play a significant role in insulin vesicle exocytosis in mouse [62–64] or rat [61,65] β-cells. However, here we show that Ca_V_2.1 is the main isoform expressed in neonatal β-cells. Although we did not measure the contribution of Ca_V_2.1 to vesicle exocytosis, we postulate that in early mouse developmental stages Ca_V_2.1 channels occupy a prominent role in vesicle exocytosis similar to what has been shown in human β-cells [20] or mouse and human α-cells [66–70].

The decrease in T-type currents is consistent with the functional shift from cell expansion toward stimulus-secretion coupling. In rodent β-cells, T-type currents are largely a hallmark of the immature or the diseased β-cell rather than of the healthy adult β-cell. Ca_V_3.1 is absent from healthy adult mouse β-cells and has only a minor current contribution in healthy rat β-cells consistent with the ontogenetic downregulation of Ca_V_3 channels during β-cell maturation [18,71]. The channels expression increases under pathophysiological conditions and has been linked to β-cell apoptosis [17,72]. Additionally, Ca_V_3.1 expression is by itself sufficient to impair insulin release and glucose homeostasis through a calcineurin–FoxO1 pathway that downregulates the exocytotic machinery [18]. Functionally, the channel operates upstream of exocytosis, as a low-threshold depolarizing conductance [73–76] that sets β-cell excitability rather than directly controlling vesicle release. In INS-1 cells, the T-type blocker Ni^2+^ abolishes the low-threshold depolarization, prolongs action-potential latency, lowers firing frequency, and reduces glucose-stimulated insulin secretion in a dose-dependent manner [77]. Similarly, the T-type blocker NNC 55–0396 suppresses the membrane-potential and cytosolic calcium oscillations that drive exocytosis [78]. These reports therefore reinforce the notion that T-type channel expression in neonatal β-cells can generate the few APs on top of the depolarizing plateaus. However, because of the rapid T-type channel inactivation [79] combined with limited L-type current density, the electrical activity terminates fast despite sustained depolarized plateau potentials. In contrast, β-cells from 14-day-old and adult mice displayed the characteristic repetitive AP-trains that lengthened with increasing glucose concentrations. Correlating with the larger calcium current density, the 14-day-old β-cells were characterized by lower glucose threshold compared with adults, consistent with prior evidence that the glucose threshold for β-cell activation increases during postnatal maturation [8]. K_ATP_ current amplitude and their glucose-induced inhibition were comparable in 14-day-old and adult β-cells, indicating that the observed differences in excitability do not arise primarily from altered K_ATP_ channel function [14,45] but rather changed calcium current amplitude and different isoform expression.

However, the insulin release does not directly correlate with β-cell calcium influx amplitude or glucose-dependent electrical activity. Due to islet size limitations, dynamic insulin release measurements from neonatal islets were technically not possible. However, it has previously been shown that vesicle exocytosis evoked by a train of depolarizing steps could be detected in only a subset of neonatal β-cells, and was substantially smaller than that recorded two days later [80]. Considering that the plateau potential was similar to the depolarization threshold of the high-voltage-gated calcium currents we can speculate that neonatal β-cells have a continuous low level insulin release caused by a small but constant calcium influx in the absence of APs and independent of the glucose levels. In β-cells and islets of 14-day-old mice, the larger calcium influx amplitude correlates with a better glucose-induced electrical activity, but the insulin release does not. The maximal Ca_V_ current density at postnatal day 14, elevated glucose sensitivity, but reduced insulin release suggest that this intermediate developmental stage might represent a transitional state in β-cell functional maturation. Although the calcium current density (per unit capacitance) is largest in juvenile β-cells, the larger size of adult β-cells means that the absolute peak calcium current is comparable between juveniles and adults. The lower insulin output of juvenile islets therefore cannot be explained by reduced total calcium entry and instead reflects the lower insulin content and the smaller releasable pool downstream of calcium influx. We did not quantify insulin transcript levels. However, the ~3-fold higher total insulin content of adult islets (Figure 5(F)) is the functionally relevant variable and is consistent with the documented postnatal up-regulation of insulin biosynthesis and granule biogenesis [3,5,8,10,81,82]. Further supporting this notion comes from the β-cell exocytosis measurements, which revealed significantly smaller cumulative increases in juveniles compared with adults. This could reflect an ongoing reorganization of exocytotic proteins and calcium channel–SNARE coupling. Supporting this hypothesis, previous studies have demonstrated that maturation of the exocytotic machinery involves changes in synaptotagmin expression and calcium sensitivity of vesicle fusion [4,10], processes likely coordinated with the observed shifts in Ca_V_ channel composition [18]. Supporting this observation, our data show that despite higher Ca_V_ currents in 14-day-old β-cells, the RRP release is similar to adult β-cells. Another contributing factor to the lower insulin release is the 3-fold lower total islet insulin content in the juvenile islets compared to adults. These findings indicate that maturation of insulin release might involve both improved coupling between calcium influx and vesicle exocytosis as well as increased granule content, as previously reported [9,10].

The progressive increase in glucose threshold from 2.1 mM in neonates, to 6.1 mM in juvenile, and to 8.9 mM in adult β-cells likely reflects adaptation to development and changing metabolic demands. During early life, lower glucose thresholds ensure insulin release critical for organism development even at lower carbohydrate intake. As the animal transitions to independent feeding, higher thresholds help prevent hyperinsulinemia at normoglycemic levels. These findings are consistent with observations in humans in which neonatal β-cells exhibit lower glucose responsiveness and incomplete electrical activity [6,7]. Thus, the developmental dynamics of Ca_V_ channel composition identified here in mice may have direct translational relevance for understanding human β-cell maturation.

Additionally, understanding the role of Ca_V_ channel isoform expression on β-cell maturation is critical for improving stem cell–derived β-cell replacement therapies. Current differentiation protocols often generate β-like cells with immature electrophysiological signatures, including persistent T-type calcium currents and low glucose thresholds [71,83–85]. Our results suggest that promoting a developmental transition toward dominant L-type channel expression could enhance functional maturation and glucose responsiveness of engineered β-cells.

Our study has several limitations. First, paracrine modulation [86–89] of β-cells has not been considered, however it most likely changes substantially during postnatal development considering that the islet size and cellular composition change. Second, sex-specific effects were not evaluated during postnatal development. Previously we showed that sex is a major determinant of adult β-cell glucose-dependent excitability and insulin release, thus inclusion of both sexes across ages may uncover additional developmental changes [26]. However, the major developmentally regulated parameters that we observe here to be different (Ca_V_ isoform composition, glucose threshold, vesicle exocytosis) did not show a sexual dimorphism in adults [26]. Third, although our findings implicate Ca_V_ channels as major determinants of developmental excitability, other ion channels, such as voltage-gated potassium or sodium channels, may also undergo maturation-dependent changes influencing β-cell excitability.

In summary, in this study we demonstrate that postnatal β-cell maturation requires a sequential refinement of Ca_V_ channel isoform composition that shapes electrical activity, glucose sensitivity, and insulin secretion. The cumulative effect of enhanced β-cell mass, increased excitability, and exocytotic capacity causes a 7-fold rise in plasma insulin levels between juvenile and adult mice.

## Data Availability

The data that support the findings of this study are available from the corresponding author PT upon reasonable request.
